# Transcutaneous electrical acupoint stimulation induced sedative effects in healthy volunteers: A resting-state fMRI study

**DOI:** 10.3389/fnhum.2022.843186

**Published:** 2023-01-19

**Authors:** Zhihong Lu, Tingting Huo, Jiao Deng, Fan Guo, Kang Liu, Peng Liu, Qiang Wang, Lize Xiong

**Affiliations:** ^1^Department of Anesthesiology and Perioperative Medicine, Xijing Hospital, Fourth Military Medical University, Xi'an, China; ^2^Department of Radiology, Xijing Hospital, Fourth Military Medical University, Xi'an, China; ^3^Life Sciences Research Center, School of Life Sciences and Technology, Xidian University, Xi'an, Shaanxi, China; ^4^Translational Research Institute of Brain and Brain-Like Intelligence, Department of Anesthesiology, Shanghai Fourth People's Hospital Affiliated to Tongji University School of Medicine, Shanghai, China

**Keywords:** acupuncture, functional magnetic resonance imaging, sedation, healthy volunteer, clinical trial

## Abstract

**Background:**

Previous studies indicated the sedative effect of acupoint stimulation. However, its mechanism remains unclear. This study aimed to investigate the sedative effect of transcutaneous electrical acupoint stimulation (TEAS) and to explore the brain regions involved in this effect in healthy volunteers using functional magnetic resonance imaging (fMRI) techniques.

**Methods:**

In this randomized trial, 26 healthy volunteers were randomly assigned to the TEAS group (receiving 30 min of acupoint stimulation at HT7/PC4) and the control group. fMRI was conducted before and after the intervention. The primary outcome was the BIS value during the intervention. Secondary outcomes included the amplitude of low-frequency fluctuation (ALFF) and region of interest (ROI)-based functional connectivity (FC) showed by fMRI.

**Results:**

In healthy volunteers, compared with the control group, ALFF values in the TEAS-treated volunteers decreased in the left thalamus, right putamen, and midbrain, while they increased in the left orbitofrontal cortex. More FC existed between the thalamus and the insula, middle cingulate cortex, somatosensory cortex, amygdala, and putamen in subjects after TEAS treatment compared with subjects that received non-stimulation. In addition, ALFF values of the thalamus positively correlated with BIS in both groups.

**Conclusion:**

Transcutaneous electrical acupoint stimulation could induce a sedative effect in healthy volunteers, and inhibition of the thalamus was among its possible mechanisms.

**Clinical trial registration:**

www.ClinicalTrials.gov; identifier: NCT01896063.

## Introduction

Insomnia is a common clinical disease that may seriously affect the quality of life. Pharmaceutical interventions for insomnia can induce adverse effects, including tolerance and addiction. Acupuncture has been used as adjunctive therapy in many perioperative settings, including additive sedation (Si et al., 2009; Lu et al., 2015). Acupressure on specific acupoints like Shenmen (HT7) and Ximen (PC4) can significantly reduce stress levels and induces significant relaxation and drowsiness (Ekblom et al., 1991; Dullenkopf et al., 2004). However, whether acupoint stimulation at these specific acupoints could generate sedative effects, and the mechanism involved in this effect in healthy subjects remains unclear.

Previous studies on acupuncture and fMRI mostly focused on acupuncture analgesia (Huang et al., 2021). Less is known about the change in brain function and brain activity during sedation by acupuncture. Moreover, different brain regions were involved in different acupuncture effects. Brain regions involved in acupuncture analgesia for low back pain were reported to be mainly located in the pain matrix and descending pain modulatory system (Wen et al., 2021). At the same time, patients with migraine-induced emotional disorders showed a lower amplitude of low-frequency fluctuations (ALFFs) value in the left anterior cingulate (Zhang et al., 2021).

In the current study, we aimed to investigate the sedative effect of transcutaneous electrical acupoint stimulation (TEAS) on the level of consciousness and the brain regions involved in healthy volunteers using functional magnetic resonance imaging (fMRI).

## Materials and methods

The study was approved by the Institutional Ethics Committee (Xijing Hospital, Fourth Military Medical University, No. KY20110901-8) and registered in *Clinicaltrials.com* (NCT01896063).

### Participant's inclusion and randomization

Written informed consent was obtained from each participant before the experiment. Inclusion criteria for the volunteer included right-handedness according to the modified Edinburgh Handedness Questionnaire. Exclusion criteria included (1) abnormal sleeping habits, (2) a history of analgesics or hypnotics for longer than 3 months), (3) a history of mental, psychiatric, or neurological disorders, (4) a history of drug abuse or current use of antidepressants or hypnotics, and (5) experience of acupuncture in the last 3 months.

A total of 26 subjects were randomly assigned to the TEAS group (30 min of TEAS stimulation at bilateral HT7/PC4) or control group (electrodes were connected at the same acupoints for 30 min without stimulation) using a computer-generated random allocation sequence (*n* = 13 for each). The group allocation was sealed in an envelope and was opened by an investigator who did the intervention before the administration of the intervention. All subjects were in the supine position and underwent continuous monitoring of heart rate (HR), blood pressure (BP), oxygen saturation (finger pulse oximetry), and bispectral index (BIS). Vital signs and BIS values were recorded every 5 min during the intervention.

### Intervention: TEAS

The chosen acupoints were bilateral Shenmen (HT7)/Ximen (PC4, see Supplemental Digital Content 1) acupoints. Electrodes were attached to the skin and connected to the Hwato Electrical Acupoint Stimulator (model no. SDZ-V; Suzhou Medical Appliances Co., Ltd., Suzhou, China) (see Supplemental Digital Content 1, which shows the attachment of the electrodes and the stimulator). The device produced “disperse-dense” waves of alternating frequencies between 2 and 10 Hz. The threshold intensity was defined as the maximally tolerated intensity of the participant to the “Teh Chi” sensations of heaviness, numbness, and swelling at the point of stimulation. The parameters of stimulation are described in Supplementary Digital Content 2.

None of the subjects in the study had previously received transcutaneous electrical stimulation and were informed that they may or may not feel the electrical stimulation. Interventions were performed by a designated investigator who was not involved in the follow-up. The stimulator was placed in an opaque box.

### Functional magnetic resonance imaging

Before and after the intervention, the subjects underwent 17 min of fMRI scanning at the MR Research Center (Xijing Hospital, Airforce Military Medical University, Xi'an, Shaanxi, China). Details of fMRI are shown in Supplementary Digital Content 3.

Images were pre-processed using Statistical Parametric Mapping software (SPM8, Neurology, London, UK, http://www.fil.ion.ucl.ac.uk). The first five time points of each functional time series were discarded to allow for magnetization equilibrium. The remaining images were corrected for time delays between different slices and realigned to the first volume. Head motion parameters were computed by estimating the translation in each direction and the angular rotation on each axis for each volume. If the head motion parameters exceeded 1.5 mm or 1.5°, the subject was excluded from further analysis. Structural images of each subject were then co-registered with the functional images. The co-registered T1 images were segmented into gray matter, white matter, and cerebrospinal fluid. The nonlinear transformations from native space to standard space were obtained from the co-registration of the T1 images with the normalized Montreal Neurological Institute (MNI) template. The functional images were transformed into standard space using the same normalization parameters of T1 images and re-sampled to 3 × 3 × 3 mm voxels. The normalized functional images were spatially smoothed with a 6-mm full-width at half-maximum (FWHM). Gaussian Kernel and linear trends were removed. Temporal bandpass (0.01–0.08 Hz) filtering was performed to remove low-frequency drifts and high-frequency physiological noise (Cordes et al., 2001).

The amplitude of low-frequency fluctuation evaluation demonstrates low-frequency blood oxygen level-dependent (BOLD) signal fluctuation and the intensity of regional spontaneous brain activity, which is important for the detection of functional connectivity in central processing. ALFF was acquired using REST software (www.restfmri.net; Yan and Zang, 2010). For each voxel, time series were transformed to the frequency domain using fast Fourier transformation. The square root of the power spectrum was computed and then averaged across a predefined frequency interval at each voxel in the frequency range. The averaged square root of the power spectrum was considered ALFF, which is the strength of the low-frequency oscillators (Zang et al., 2007).

The mask of the region of interest (ROI) was created by a 6-mm sphere, and the central coordinates were located in the peak of the significant activation/deactivation based on differences between the groups. The mean BOLD time course was then extracted from the ROI as a regressor in functional correlation analysis. Correlation maps were created by computing the correlation coefficients between the BOLD time course from the ROI and the BOLD time course from all other brain voxels. Finally, correlation coefficients were converted to an approximately normal distribution using Fisher's *z*-transformation.

### Outcome measures

The primary outcome was BIS value during the intervention. As shown by fMRI, secondary outcomes included ALFF and ROI-based functional connectivity (FC).

### Statistical analysis

The trial was explorative, and a small sample of 26 participants was used. Statistical analysis was performed with SPSS software (version 19.0, SPSS Inc^®^, Chicago, IL, USA). A *p*-value of < 0.05 was considered statistically significant. Demographic data were analyzed using ANOVA and the chi-square test. For BIS values, ANOVA and Dunnett's tests were used. The data were presented as mean ±SD, median (interquartile range), or percentages. For imaging analysis, paired *t*-test was applied to assess ALFF-related differences at the second-level analysis. One-sample *t*-test was then used to measure the group-level connectivity related to the ROI. The thresholds of the contrasts were set as *P* < 0.05 [false-discovery rate corrected (FDR corrected)], and the cluster size was set as >5 voxels. Age and sex were considered covariates of no interest.

## Results

**Figure 1:**
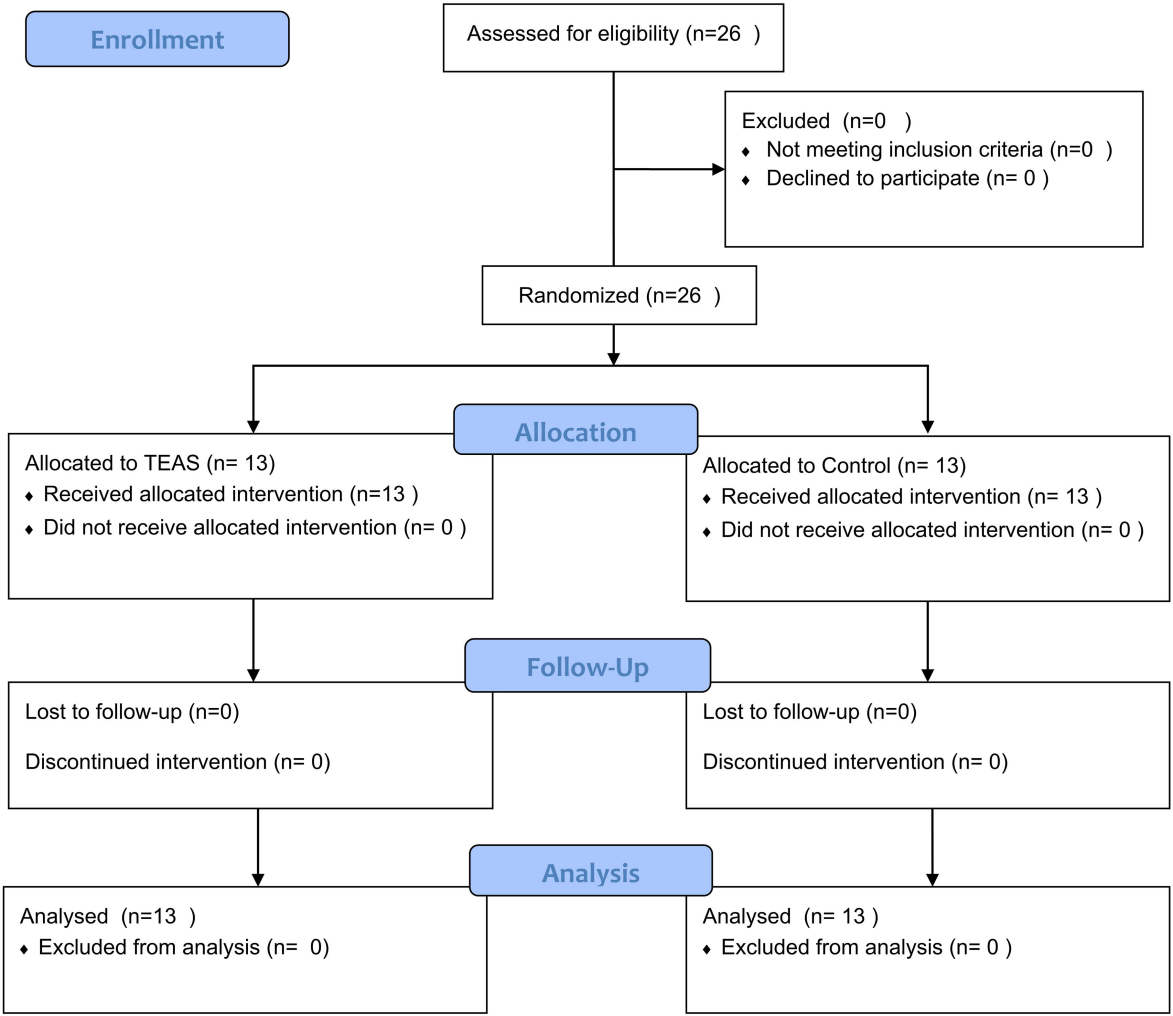
Flowchart of participant's enrollment.

**Figure 2:**
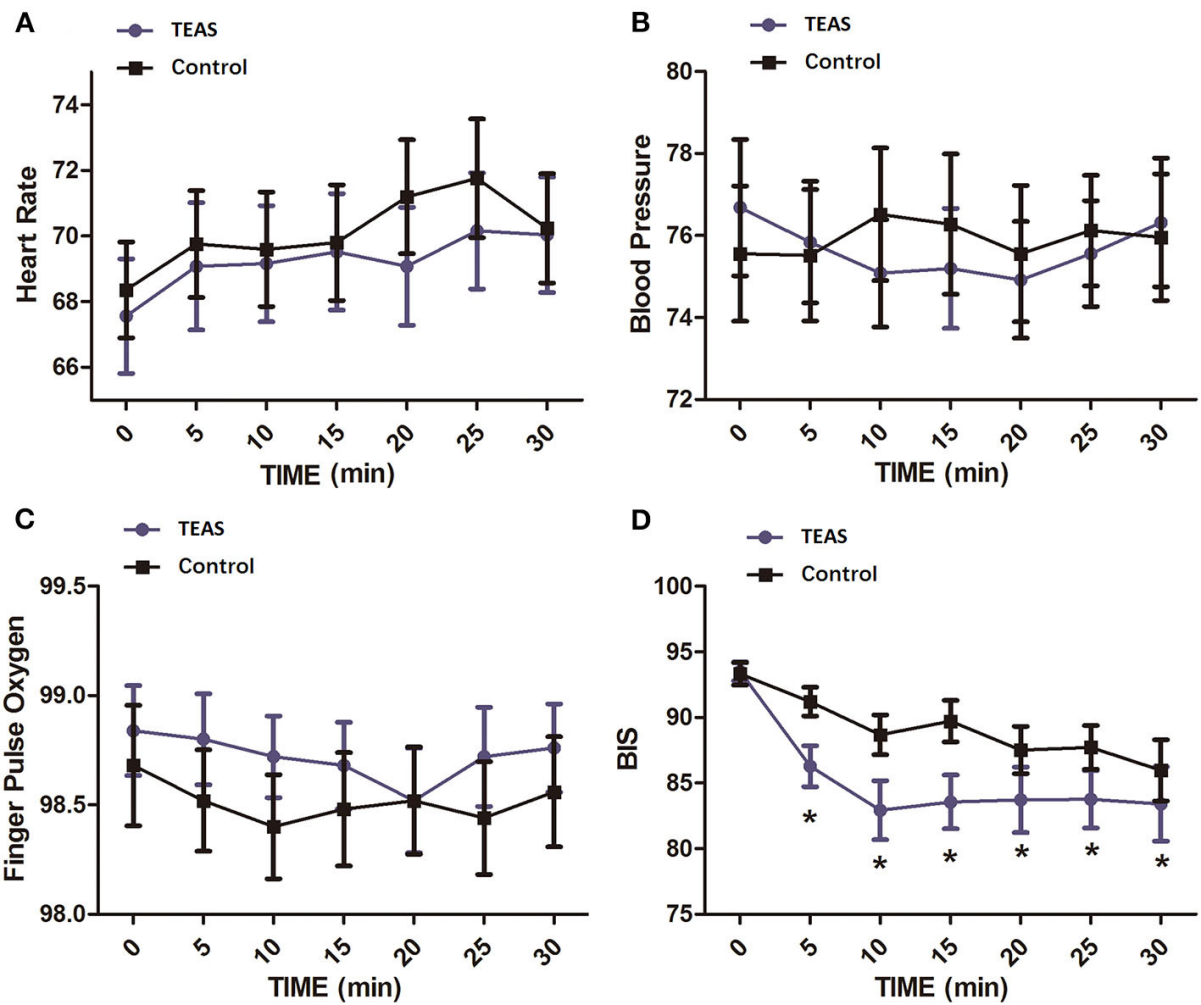
Vital signs and BIS values at different time points in healthy volunteers of the TEAS group and the control group. **(A)** Heart rate; **(B)** mean blood pressure; **(C)** pulse oxygen saturation; **(D)** BIS. The mean BIS values were significantly decreased in the TEAS group at 5, 10, 15, 20, 25, and 30 min after TEAS treatment compared with the control group. *P < 0.05. TEAS, transcutaneous electrical acupoint stimulation; BIS, bispectral index.

A total of 26 healthy volunteers, including 12 men and 14 women, were enrolled. All subjects completed the study (*n* = 13 per group, Figure 1). There were no significant differences among the groups with respect to demographic parameters (Table 1). Subjects tolerated the treatment well, and no adverse event was reported. Heart rate, mean blood pressure, and SpO_2_ at different time points exhibited no difference between the two groups (Figures 2A–C).

**Table 1:** Demographic parameters and baseline vital signs of the participants.

	**TEAS (*n* = 13)**	**Control (*n* = 13)**	***P*-value**
Gender (M/F)	7/6	5/8	0.82
BMI (kg·m^−2^)	22.4 ± 2.2	23.0 ± 2.6	0.67
Heart rate (bpm)	62 (14)	65 (12)	0.78
Mean blood pressure (mmHg)	70.1 ± 11.7	68.2 ± 18.3	0.60
SpO_2_ (%)	100 (1.0)	100 (1.2)	0.93

Values are mean ± SD, median (interquartile range), or the number of participants. TEAS, transcutaneous electrical acupoint stimulation; BMI, body mass index; SD, standard deviation.

### BIS values after TEAS treatment

The baseline BIS values were not different between the two groups. BIS values in each group significantly changed with time. At time points after TEAS began, the BIS value was significantly lower in the TEAS group than in the control group (Figure 2D).

### Amplitude of low-frequency fluctuation

**Figure 3:**
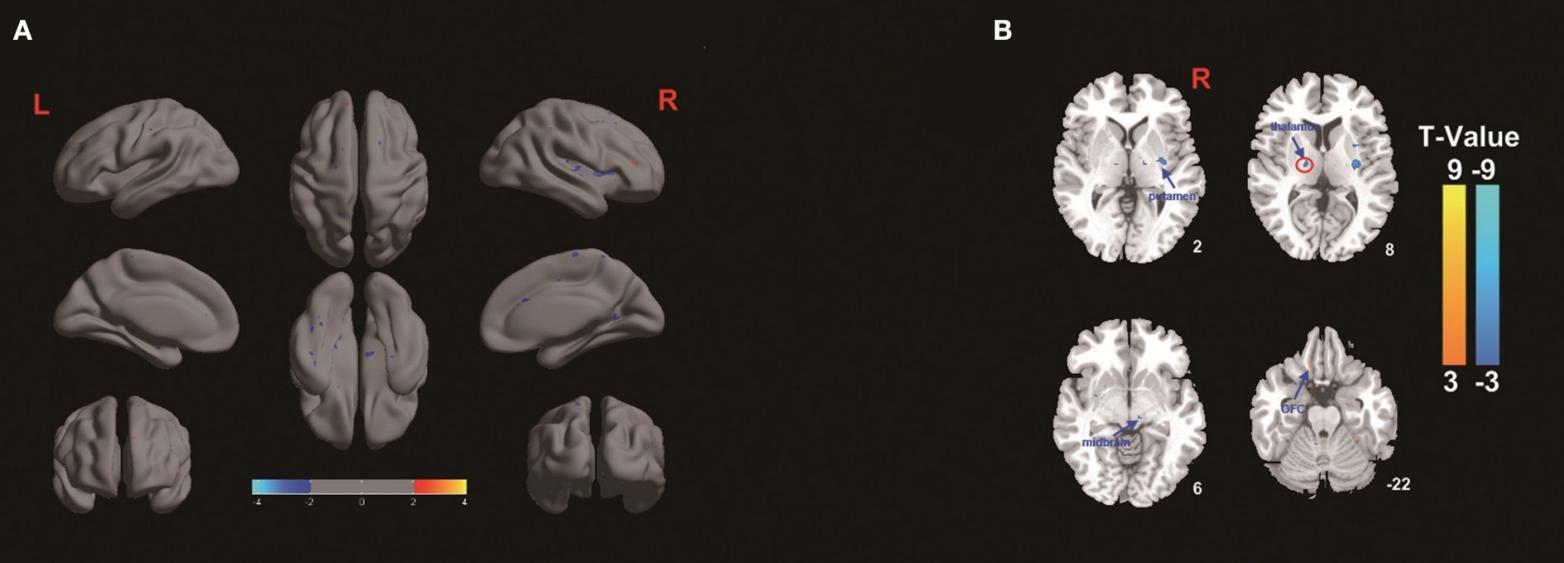
**(A,B)** ALFF differences in healthy volunteers between the TEAS group and the control group. The warm color shows increased ALFF values in the OFC in the TEAS group, while the cold color shows decreased ALFF values in the left thalamus, right putamen, and midbrain in the TEAS group. F, The amplitude of low-frequency fluctuation; OFC, orbitofrontal cortex; TEAS: transcutaneous electrical acupoint stimulation.

**Figure 4:**
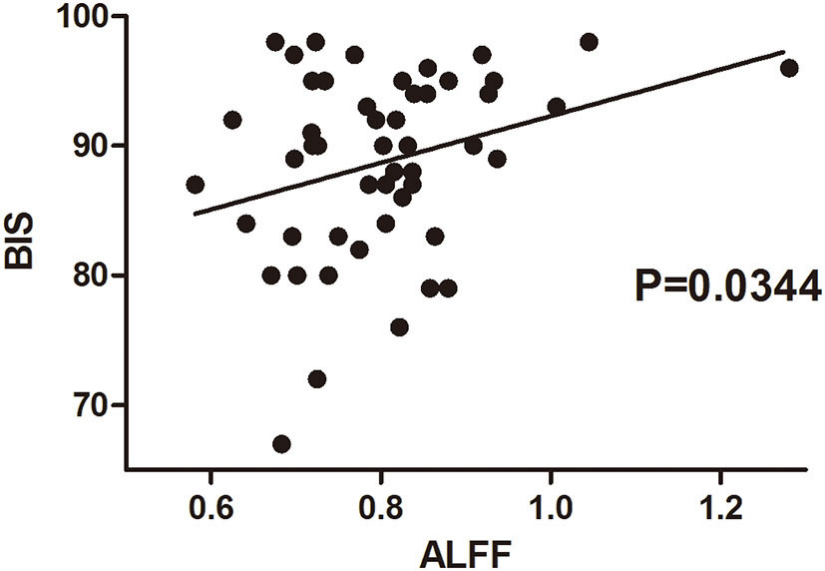
Correlation between BIS and ALFF values of the thalamus in healthy volunteers of both groups before and after the intervention. The correlation between BIS values at the beginning and by the end of the intervention in each volunteer was evaluated with their averaged ALFF values from the fMRI scan before and after the intervention. There was a significantly positive correlation between the average ALFF values of the thalamus and BIS values. BIS, bispectral index; ALFF, amplitude of low-frequency fluctuation.

The amplitude of low-frequency fluctuation was used to evaluate regional spontaneous neuronal activity on fMRI. The ALFF changes in the TEAS group compared with the control group are shown in Figure 3. The left thalamus in the TEAS group showed a significantly lower ALFF value than the control group; in addition, several other neural regions, such as the right putamen and midbrain, exhibited lower ALFF values than the control group, whereas the left orbitofrontal cortex (OFC) showed increased ALFF values compared with the control group (Figure 3). Correlation analysis indicated a significant positive correlation between the average ALFF values of the thalamus and BIS (*r*^2^ = 0.0899, *P* = 0.03, Bonferroni corrected; Figure 4).

### Functional connectivity

**Figure 5:**
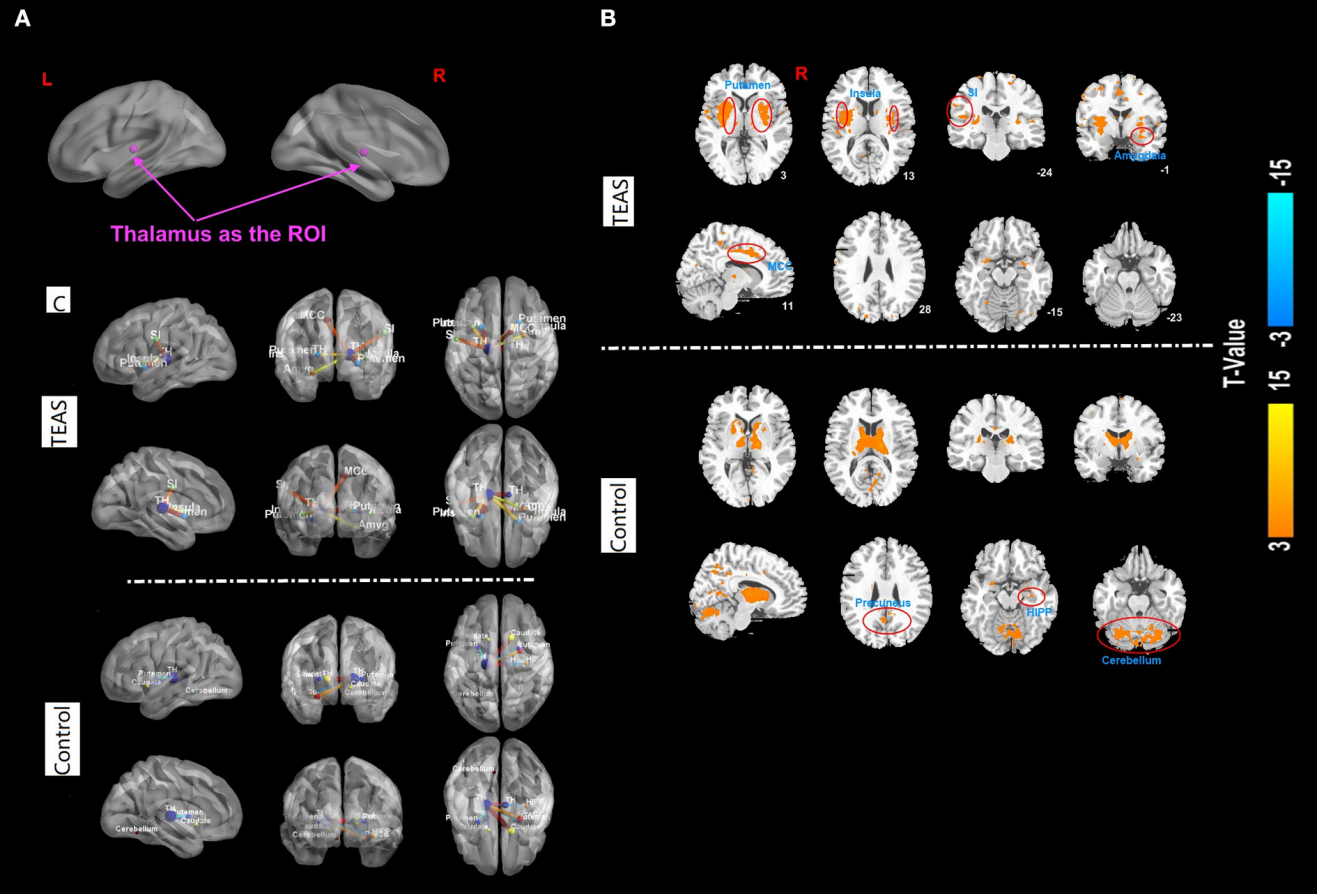
**(A–C)** Functional correlation map associated with the ROI in the two groups after TEAS stimulation or control intervention. The warm color shows the positive functional connectivity between the ROI (thalamus) and other regions. The cold color shows negative functional connectivity between the ROI and other regions. Significant positive connectivity was found between the thalamus and bilateral insula, bilateral putamen, left primary SI, right MCC, and right amygdala in the TEAS group. Meanwhile, significant connectivity was detected between the thalamus and the bilateral precuneus, right hippocampus, and bilateral cerebellum in the control group, indicated by orange circles. ROI, region of interest; TEAS, transcutaneous electrical acupoint stimulation; SI, somatosensory cortex; MCC, middle cingulate cortex.

The results of functional connectivity showed significant positive connectivity between the thalamus and bilateral insula, bilateral putamen, left primary somatosensory cortex (SI), right middle cingulate cortex (MCC), and right amygdala in the TEAS group. Meanwhile, significant connectivity was detected between the thalamus and the bilateral precuneus, right hippocampus, and bilateral cerebellum in the control group (Figure 5).

## Discussion

The results of the present study indicated that (1) TEAS significantly decreased the BIS values in healthy volunteers and (2) TEAS decreased regional brain activity primarily in the left thalamus, right putamen, and midbrain, whereas it induced activation in the left OFC. Our findings suggested that TEAS-induced sedation may be related to altered neuronal activity, such as the inhibition of ARAS.

Advances in imaging techniques have facilitated many clinical reports regarding the involvement of brain networks in the effects of electrical acupoint stimulation. The cholinergic system has been reported to mediate electroacupuncture (EA) effects (Lin et al., 2011; Guo et al., 2012). The ascending reticular activating system (ARAS) is a network of nerve fibers ascending from the brain stem cholinergic neurons, which activates the forebrain during the state of wakefulness and rapid eye movement (REM) sleep (Brown et al., 2012). Brain stem cholinergic neurons promote cortical activation via their projections to the thalamus, comprising a major component of the dorsal ARAS pathway (Steriade et al., 1988), which plays a critical role in maintaining wakefulness (Boly et al., 2007). In the current study, we conducted fMRI in healthy volunteers before and after TEAS at bilateral HT7/PC4 or sham stimulation. ALFF of the left thalamus was found to be reduced significantly in the TEAS group. Previous fMRI study has indicated a widespread decrease in brain activity during NREM sleep, particularly in the thalamus, somatosensory, and basal ganglia (Kaufmann et al., 2006). The reduced activity in the thalamus may indicate that TEAS at bilateral HT7/PC4 generates a pro-sedative function in the brain by inhibiting the thalamus activity. ROI related to the thalamus was evaluated to identify further brain regions that are involved in the effects of TEAS at these acupoints.

Transcutaneous electrical acupoint stimulation also resulted in stronger activation of the left OFC area compared with the control group. The OFC is a structure in which multiple sensory information and reward information converge. It modulates rewards and punishment. Activation of the OFC in humans can be produced by pleasant touch to the hand or by pain (Rolls, 2004). Since no complaints of pain were reported by the volunteers undergoing TEAS, the increased ALFF of OFC in TEAS groups might be induced by pleasant feelings generated by the mild stimulation at HT7/PC4 acupoints. Other regions that showed reduced ALFF was the right putamen and the midbrain. The putamen is traditionally identified as a region that facilitates limb movements. It is the primary site for the principal neuropathology associated with Parkinson's disease (Aminoff, 2001), which contains mainly dopaminergic neurons. Interestingly, the dopaminergic system has been recently indicated in the mechanism of general anesthesia and sleep (Taylor et al., 2016; Meloni et al., 2020), especially a specific reduction in activity was noted in the putamen after propofol anesthesia (Mhuircheartaigh et al., 2010). The ALFF reduction after TEAS observed in the volunteers may also be a sedative-like fMRI manifestation. Other studies observed ipsilateral putamen activation after GB34 acupuncture stimulation in healthy participants (Yeo et al., 2016) and patients with PD (Yeo et al., 2014), indicating acupoint-specific effects on the central nervous system. Acupoint selection is very important in generating desirable effects. On this note, a recent trial that evaluated whether electroacupuncture could reduce sedation requirements during colonoscopy selected ST36, PC6, and LI4 as target acupoints (Yeo et al., 2014). The negative results generated from that trial when compared to the effectiveness of our study rely at least partly on the difference in acupoint selection.

The fMRI result in our study demonstrated a reduced spontaneous activity around the location of the red nucleus of volunteers after TEAS. The red nucleus plays an important role in motor control and locomotion. Reduced activity in this nucleus may be due to a reduced requirement of activity for motor control, which indicated that volunteers in this group might be in a more sedative state than subjects in the control group. Chen et al.'s study using PET imaging demonstrated increased metabolism at the red nucleus after stimulating TE5 (Chen et al., 2012; Yang et al., 2013), which is considered responsible for moving energy between the upper body and the lower body. Another study using fMRI also demonstrated increased activity in the red nucleus after ST36 stimulation compared with sham stimulation (Napadow et al., 2009). These differences, again, may be attributed to the fact that different acupoints were selected.

In the TEAS group, decreased ALFF values were found in the thalamus, while increased functional connectivity was found between the thalamus and bilateral insula, bilateral putamen, left SI, right MCC, and right amygdala. These brain areas are involved in sleep disorders, memory, mood/motivation regulation, and depression (Phelps and LeDoux, 2005; Ramel et al., 2007). Decreased functional connectivity between the thalamus and amygdala was reported in patients with insomnia (Huang et al., 2012). In our study, increased functional connectivity between these two nuclei may be a sign of a sedative effect. Increased functional activity between the thalamus and these brain regions may indicate more subcortical modulation of brain activities in these regions by the thalamus, generating sleepiness, comfort, and calm feelings that are all associated with pro-sedative effects. In contrast, control subjects revealed increased thalamic connectivity to brain regions, including the bilateral precuneus, right hippocampus, and bilateral cerebellum. These brain regions are mainly involved in emotion and activity during wakefulness. Precuneus connectivity was found to be significantly stronger in conscious patients than those in unconscious patients (Vanhaudenhuyse et al., 2010). These results also indicate that TEAS at HT7/PC4 affects brain activity in a pro-sedative way.

A significant positive correlation was found between the average ALFF values in the thalamus on fMRI and BIS values. The bispectral index (BIS) was developed to quantify the depth of general anesthesia (Punjasawadwong et al., 2014) and sedation (Herzog et al., 2021). It has been reported to be used as a simplified tool to evaluate the depth of natural sleep (Benissa et al., 2016). It has been suggested that a positive correlation between the scalp EEG and deep brain nuclei reflected a mechanism of cortical activity modulation exerted by the thalamus (Fukunaga et al., 2006). Reduced thalamus activity observed after TEAS in volunteers is associated with their lower BIS value, which further corroborates that TEAS may reduce the level of consciousness through the inhibition of the thalamus activity.

Our study has several limitations. First, the number of subjects was relatively small, which may lower the statistical power. Second, we observed the sedative effect using the BIS value instead of sedation scores, such as the Richmond agitation-sedation scale. Third, it was difficult to blind the subjects, as the intervention started when the subjects were awake. To minimize possible bias, we selected participants with no experience with electrical stimulation and informed them that they may or may not feel the stimulation. The electrodes were placed similarly for all groups, and the intensity threshold was testified for all participants. The stimulator was placed in an opaque box.

## Conclusion

Transcutaneous electrical acupoint stimulation at HT7/PC4 reduced the level of consciousness in healthy volunteers, as indicated by BIS. This sedative effect may be related to modulating the function of deep brain areas, including the thalamus, putamen, midbrain, and OFC. It also generates more connectivity between the thalamus and deep brain areas involved in sleep, mood, and memory.

## Data availability statement

The raw data supporting the conclusions of this article will be made available by the authors, without undue reservation.

## Ethics statement

The studies involving human participants were reviewed and approved by the Xijing Hospital, Fourth Military Medical University. The patients/participants provided their written informed consent to participate in this study.

## Author contributions

ZL, QW, and LX: conception and study design. TH, FG, and KL: data collection or acquisition. PL and JD: statistical analysis. ZL and JD: drafted the manuscript work. LX: revising the manuscript critically for important intellectual content. All authors contributed to the article and approved the submitted version.
