# Supplementary material for: Transcutaneous electrical acupoint stimulation induced sedative effects in healthy volunteers: A resting-state fMRI study
Source: Front Hum Neurosci. 2023 Jan 19;16:843186. doi: 10.3389/fnhum.2022.843186 (PMC9893780; doi:10.3389/fnhum.2022.843186)
Supplement: Supplementary file 1 [file Data_Sheet_1.pdf]

Supplemental digital contents

Index

Supplemental digital content 1. Attachment of electrodes and stimulator

Supplemental digital content 2. Parameters of electrical stimulation

Supplemental digital content 3. Details of fMRI scanning

Supplemental digital content 1. Attachment of electrodes and connection of stimulator

Shenmen (HT7): located at the wrist crease on the radial side of the flexor carpi ulnaris tendon, between the ulna and the pisiform bones)

Ximen (PC4): located between the flexor carpi radialis tendon and the tendon palmaris longus 5 inches (Chinese inch, *cun*) above the wrist stripe

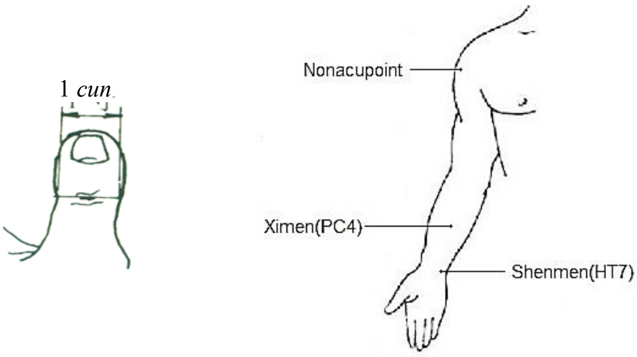

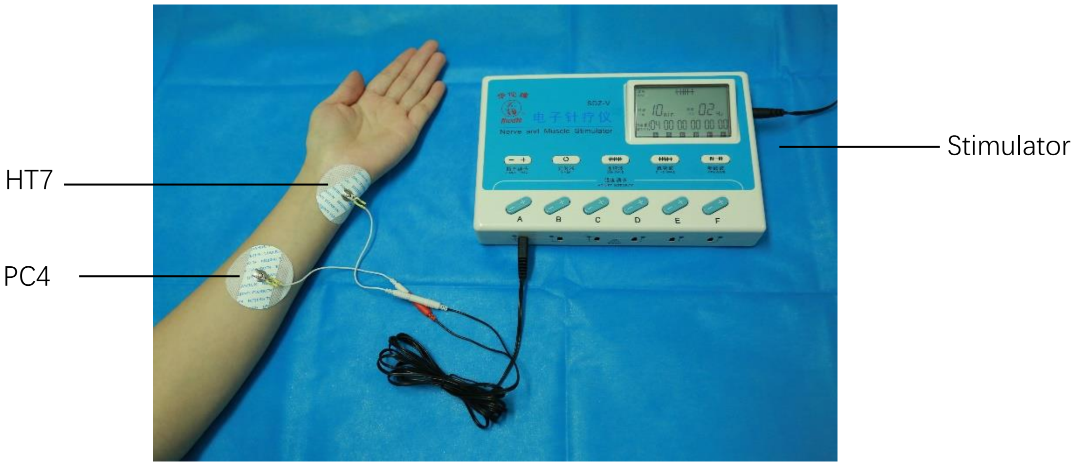

Supplemental digital content 2. Parameters of electrical stimulation

Parameters of stimulation	Group TEAS (n=13)	Group Control (n=13)
Electrodes attached to	Bilateral HT7/PC4	Bilateral HT7/PC4
Frequency	2/10 Hz	2/10 Hz
Intensity(mA)	[#]4.1 (3.3, 5.2)	0
Duration	30 min	30 min

[#]Data was shown in median (1st quartile, 3rd quartile)

TEAS: transcutaneous electrical acupoint stimulation

Supplemental digital content 3. Details of fMRI scanning

The images of each subject were acquired using a 3T Siemens scanner (Allegra, Siemens Medical System). Functional images were acquired using an echo-planar imaging (EPI) sequence with TR/TE: 2000 ms/30 ms, field of view: 240 mm×240 mm, matrix size: 64× 64, flip angle: 90°, in-plane resolution: 3.75 mm×3.75 mm, slice thickness: 5mm thick with no gaps and 30 axial slices. High resolution T1-weighted images were then collected with a volumetric three-dimensional spoiled gradient recall sequence (TR/TE: 1900 ms/2.26 ms, field of view: 240 mm×240 mm, matrix size: 240×240, flip angle=9°, in-plane resolution: 1 mm×1 mm, slice thickness=1 mm, 176 sagittal slices). During scanning, subjects were asked to close eyes and keep awake, to lie in a comfortable position and avoid body movement. A standard birdcage head coil along with a restraining foam pad was used to minimize head motion. The ears were plugged with cotton balls to reduce audio stimulations.
